# Towards tellurium-free thermoelectric modules for power generation from low-grade heat

**DOI:** 10.1038/s41467-021-21391-1

**Published:** 2021-02-18

**Authors:** Pingjun Ying, Ran He, Jun Mao, Qihao Zhang, Heiko Reith, Jiehe Sui, Zhifeng Ren, Kornelius Nielsch, Gabi Schierning

**Affiliations:** 1 Leibniz Institute for Solid State and Materials Research, Dresden, Germany; 2 Department of Physics and Texas Center for Superconductivity at the University of Houston (TcSUH), University of Houston, Houston, TX USA; 3 National Key Laboratory for Precision Hot Processing of Metals, School of Materials Science and Engineering, Harbin Institute of Technology, Harbin, China; 4 Institute of Applied Physics, Technical University of Dresden, Dresden, Germany; 5 Institute of Materials Science, Technical University of Dresden, Dresden, Germany; 6 Department of Physics, Experimental Physics, Bielefeld University, Bielefeld, Germany

**Keywords:** Thermoelectric devices and materials, Electronic properties and materials

## Abstract

Thermoelectric technology converts heat into electricity directly and is a promising source of clean electricity. Commercial thermoelectric modules have relied on Bi_2_Te_3_-based compounds because of their unparalleled thermoelectric properties at temperatures associated with low-grade heat (<550 K). However, the scarcity of elemental Te greatly limits the applicability of such modules. Here we report the performance of thermoelectric modules assembled from Bi_2_Te_3_-substitute compounds, including p-type MgAgSb and n-type Mg_3_(Sb,Bi)_2_, by using a simple, versatile, and thus scalable processing routine. For a temperature difference of ~250 K, whereas a single-stage module displayed a conversion efficiency of ~6.5%, a module using segmented n-type legs displayed a record efficiency of ~7.0% that is comparable to the state-of-the-art Bi_2_Te_3_-based thermoelectric modules. Our work demonstrates the feasibility and scalability of high-performance thermoelectric modules based on sustainable elements for recovering low-grade heat.

## Abstract (web summary)

Though earth abundant magnesium-based materials are attractive for thermoelectrics (TEs) due to their device-level performance, realizing efficient modules remains a challenge. Here, the authors report a scalable route to realizing Mg-based compounds for high performance TE modules.

## Introduction

More than 60% of the energy generated by burning fossil fuels is dissipated as waste heat, of which more than half is low-grade heat with temperatures <550 K^1,2^. Effective harnessing this “cooler” heat to generate electricity is vital for alleviating the burden on the energy supply and reducing the emission of greenhouse gases. Although potential technologies, such as the organic Rankine cycle^3,4^, thermogalvanic cells^5^, and thermo-osmotics^6^ are being explored, these are limited by their low efficiencies, short lifetimes, and difficulty in system integration. In comparison, thermoelectric (TE) technology stands out owing to its solid-state nature, which guarantees ultra-long operational lifetime, and is particularly attractive for heat-to-electricity conversion^7^. The broader applicability of TE technology relies on the availability of high-performance materials and modules that operate efficiently below 550 K.

The energy conversion efficiency of a TE material is governed by the dimensionless figure of merit *zT*, defined as *zT* = *S*^2^*σT*/*κ*_tot_, where *S*, *σ*, *T*, and *κ*_tot_ are the Seebeck coefficient, the electrical conductivity, the absolute temperature, and the total thermal conductivity, respectively. Among various materials tested to date, Bi_2_Te_3_-based materials have unparalleled TE properties and have thus been the focus of laboratory-scale demonstrations and commercial devices that operate below 550 K with typical conversion efficiencies of about 3–6%^8–10^. However, the wider applicability of Bi_2_Te_3_-based commercial modules is severely limited by the scarcity of Te with a concentration of <0.001 ppm in the Earth’s crust^11^ and an annual production of less than 500 metric tons^12^. Therefore, it is imperative to develop TE modules from other, more abundant materials while retaining high performance at temperatures below 550 K.

In recent years, Mg-based materials, including n-type Mg_3_(Sb,Bi)_2_^13–17^ and p-type MgAgSb^18–21^ have attracted great attention from the TE community because of the nontoxic nature, abundance of their constituent elements, and their high *zT* of ~1.0 at temperatures <550 K. Moreover, these materials exhibit excellent mechanical robustness and compatible TE properties between the n-type and p-type TE materials^22,23^. Previous reports showed excellent performances of these materials at the device level. For example, Kraemer, et al. reported a ~8.5% efficiency of single-leg MgAgSb operating between 293 K and 518 K^24^. Mao, et al. improved the cooling performance by using Mg_3_(Sb,Bi)_2_ to replace the Bi_2_(Te,Se)_3_ for n-type legs^17^. These work merit the great potential of these materials in replacing the Bi_2_Te_3_ for low-grade heat recovery applications. However, for successful delivery, it is essential to employ synthesis routines that are potentially scalable for these Te-free TE modules, as well as to address their device-level issues such as geometry optimization, brazing process, and contact optimization, etc.^23^. Till now, despite their promise, the assembly of these substitute compounds into power-generation modules has not been reported.

Herein, we synthesized p-type MgAgSb and n-type Mg_3_(Sb,Bi)_2_ compounds using direct mechanical alloying followed by rapid current-assisted sintering. Note that for n-type Mg_3_(Sb,Bi)_2_, we used less than 0.2% Te as the dopant in this work to secure the material properties since its performance was widely validated, so that the module is not completely free of Te. However, Te is not an essential dopant with available alternatives, such as Sc, Nd, Y, etc., yielded similar TE performances according to several recent studies^25–27^. We reproduced these high-performance materials with a synthesis routine that is potentially scalable. Such up-scaling potential is critically important for heat-recovery applications. Subsequently, these high-*zT* compounds were translated into high-performance TE modules. We realized a high conversion efficiency of ~6.5% and ~7.0% under a temperature difference of ~250 K in a single-stage module and a segmented module, respectively. Our efficiency is comparable to those reported for Bi_2_Te_3_-based modules. This work marks a feasible, sustainable alternative to Bi_2_Te_3_-based TE modules and will spur the application of TE technology in converting low-grade heat to electricity.

## Results

### Scalable preparation of thermoelectric materials and modules

High-performance TE materials with simple synthesis are favored for module fabrication. However, the synthesis of Mg-based compounds usually involves procedures that are either complicated, expensive, or time-consuming. For example, the synthesis of Mg_3_(Sb,Bi)_2_ compounds usually involves complicated processing routines including melting (such as arc melting, induction melting, or traditional melting), pre-annealing, powerization, sintering (such as spark plasma sintering, hot pressing, or induction pressing), and post-annealing^17,28–31^. In another example, MgAgSb, being in α phase at room temperature, changes to the β phase at ~573 K, and to the γ phase at ~633 K^18^. Whereas only the α phase has the requisite high *zT*, phase-pure α-MgAgSb is difficult to obtain using traditional melting techniques unless a time-consuming annealing process is followed^20^.

**Fig. 1:**
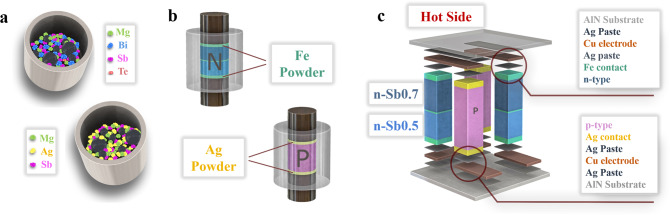
Fabrication of Te-free TE materials and modules. **a** Element weighing and mechanical alloying to prepare powder of the n- and p-type TE compounds. **b** One-step spark plasma sintering of materials and contact layers to prepare the TE legs. **c** Assembling of the Te-free TE module, including polishing, cutting, loading, positioning, and brazing the TE legs to the pre-circuited AlN ceramic plates.

An alternative synthesis routing were reported to overcome such limitations for synthesizing the n- and p-type legs using only three steps: weighing, mechanical alloying, and rapid sintering (Fig. 1a, b; “Methods”)^17,21^. Following these reports, we here employed mechanical alloying not only because it is a lower-cost way to realize large-scale production, but also because it allows for accurate stoichiometry that ensures high reproducibility, which is necessary to scale up production. This is especially essential for this work since the compounds studied here are rich in Mg, Bi, and Sb, which would otherwise largely evaporate if traditional melting techniques were used. The phases of the TE materials were characterized by X-ray diffraction (XRD). The XRD patterns (Supplementary Fig. 1a, b) indicated high purity for the n-type Mg_3.3_Bi_1.498_Sb_0.5_Te_0.002_ (denoted as n-Sb0.5) and Mg_3.3_Bi_1.298_Sb_0.7_Te_0.002_ (denoted as n-Sb0.7), and the p-type MgAg_0.97_Sb_0.99_ (abbreviated as “p-MgAgSb”). We then undertook scanning electron microscopy (SEM) and energy-dispersive X-ray spectroscopy (EDX) elemental mapping of n- and p-type legs with contact layers. The elemental distribution was nearly uniform (Supplementary Fig.1c, d) and we could find no obvious interaction between the TE materials and the contact layers.

Using the mechanically-alloyed powder samples, we then fabricated the TE legs for module assembly by sintering in one step the TE powder together with contact-layer powder on both sides. Note that such one-step sintering was also applied for segmented n-type legs. Herein, based on the previous reports, we selected Fe and Ag as the contact layers for the n- and p-type legs, respectively^17,24^. Finally, the sintered disks were diced and polished into the desired geometry, placed in the proper positions, and brazed to pre-circuited ceramic plates AlN in a vacuum furnace (Fig. 1c). Due to the simplicity, our approach will potentially reduce the assembling time greatly when compared to the traditional routines.

### Thermoelectric properties and module optimization

**Fig. 2:**
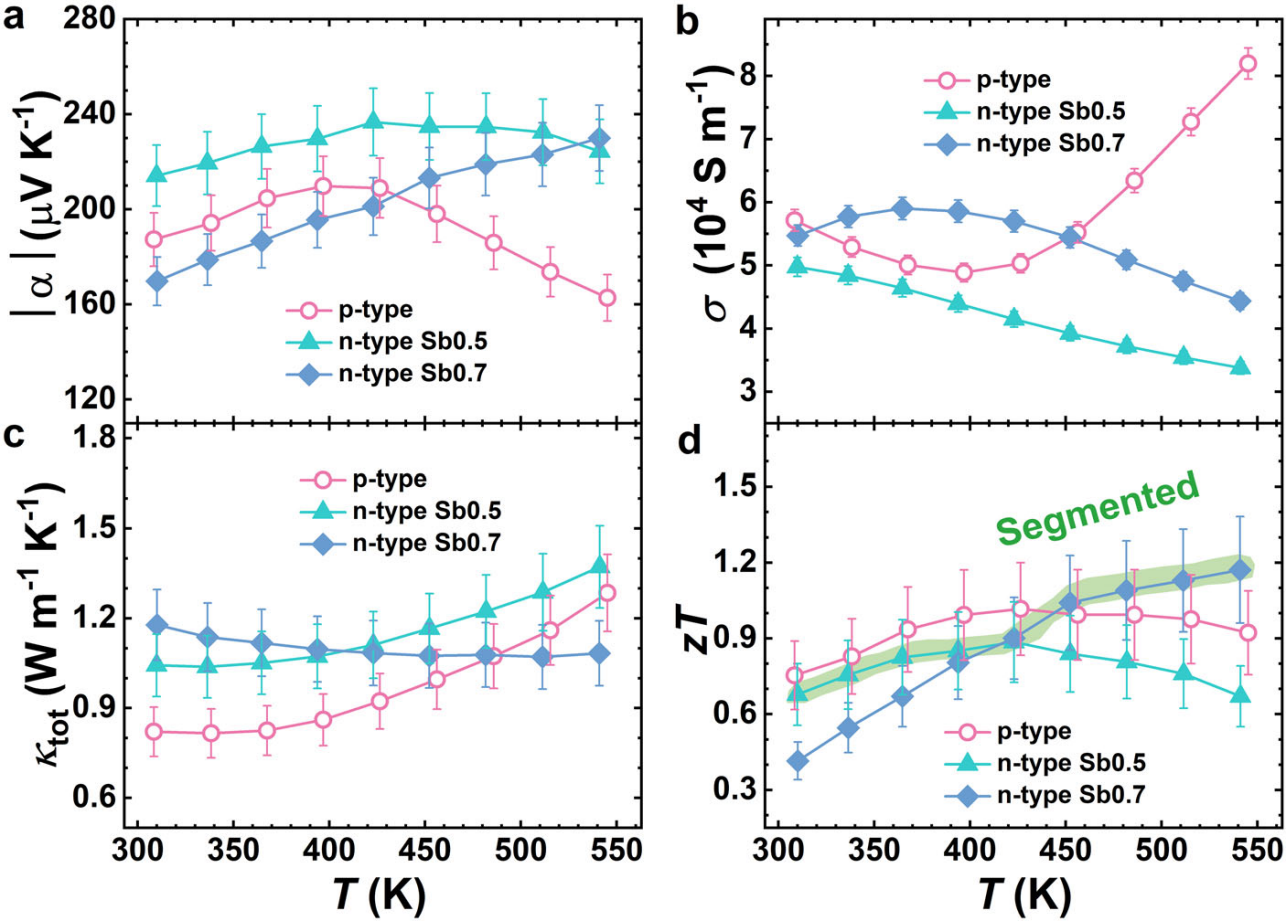
Temperature-dependent thermoelectric properties of the fabricated materials. **a** Absolute Seebeck coefficient |α|, **b** electrical conductivity *σ*, **c** total thermal conductivity *κ*_tot_, and **d** figure of merit *zT*. n-Sb0.5: Mg_3.3_Bi_1.498_Sb_0.5_Te_0.002_ (green triangles); n-Sb0.7: Mg_3.3_Bi_1.298_Sb_0.7_Te_0.002_ (blue diamonds); p-MgAgSb: MgAg_0.97_Sb_0.99_ (red rings). The error bars represent the corresponding measurement uncertainties from the commercial devices.

We measured the transport properties of the TE materials, including the electrical conductivity, the Seebeck coefficient, and the thermal conductivity. The compounds synthesized in this work, including n-Sb0.5, n-Sb0.7, and p-MgAgSb, possess similar properties when compared to previous reports (Fig. 2) despite the straight forward synthesis procedure^17,20^. For p-MgAgSb, we obtained a peak *zT* of ~1.0 at 423 K and an average *zT* of ~0.9 at temperatures ranging from room temperature to 548 K (Fig. 2d). For the n-type materials, whereas n-Sb0.5 showed a higher *zT* up to 423 K, n-Sb0.7 exhibited better performance at higher temperatures (423 K to 548 K). The peak *zT* values reach 0.9 (at 423 K) and 1.2 (at 548 K) in n-Sb0.5 and n-Sb0.7, respectively.

**Fig. 3:**
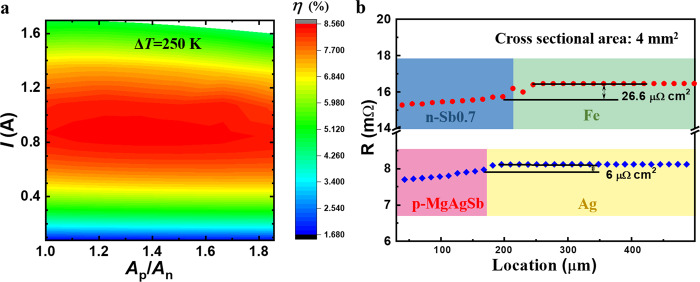
Module optimization. **a** Simulated efficiency (*η*) with respect to the *A*_p_/*A*_n_ ratio and the working current (*I*) for the single-stage module (n-Sb0.7) under *T*_hot_ = 543 K and *T*_cold_ = 293 K. **b** Measured electrical contact resistivity at the n-type/Fe and p-type/Ag junctions. The red circles and blue diamonds represent the scanning resistance across the junctions, the solid horizontal lines indicate the contact resistivity.

Based on the *zT* profiles of n-Sb0.5 and n-Sb0.7, we postulated that a segmented leg could maximize the average *zT* of the n-type materials. According to the transport properties these compounds, we employed finite element simulation to assist in designing the geometrical configuration of the TE modules. With a hot-side temperature (*T*_hot_) of 548 K and a cold-side temperature (*T*_cold_) of 293 K, we evaluated the maximum conversion efficiency as a function of the working current (*I*), the ratio of the cross-sectional areas between the p- and n-type legs (*A*_p_/*A*_n_), and the height ratio of the two n-type materials (*H*_n-Sb0.7_/*H*_n-Sb0.5_) in a segmented leg (Fig. 3a and Supplementary Fig. 2). We found that the ratio of *H*_n-Sb0.7_/*H*_n-Sb0.5_ was optimal over a large range from 0.75 to 1.75 (Supplementary Fig. 2). In addition, the ratio of *A*_p_/*A*_n_ was found to have a limited impact on efficiency (Fig. 3a). These results suggest that the module performance is not sensitive to the geometric factors, which is beneficial since it tolerates certain deviations in the TE-leg fabrication process without degrading the efficiency. Accordingly, we fabricated TE modules with segmented n-type legs with the ratio of *A*_p_/*A*_n_ being unity to facilitate the device assembling. Note that the segmented n-type legs were prepared by using the same one-step sintering routine that was used for the single-stage module (Fig. 1c) where an *H*_n-Sb0.7_/*H*_n-Sb0.5_ ratio of ~1.5 was selected. The selected *H*_n-Sb0.7_/*H*_n-Sb0.5_ ratio yielded a temperature profile that fully exploits the *zT* profiles of the n-type materials (Fig. 2d and Supplementary Fig. 2).

To further evaluate the module quality, we characterized the contact resistivity at the n-type/Fe and p-type/Ag junctions, which was found to be 26.6 µΩ∙cm^2^ and 6 µΩ∙cm^2^, respectively (Fig. 3b). Although these values remain higher than those for the benchmark Bi_2_Te_3_/Ni (1–5 µΩ∙cm^2^)^32^ that was realized upon optimizations for more than half a century, the overall interfacial resistance impacts the efficiency of our module inconsiderably. This was demonstrated by comparing the simulated internal resistance, open-circuit voltage, output powers, and conversion efficiency with and without the contact resistance of the single-stage module (Supplementary Fig. 3) and segmented module (Supplementary Fig. 4).

**Fig. 4:**
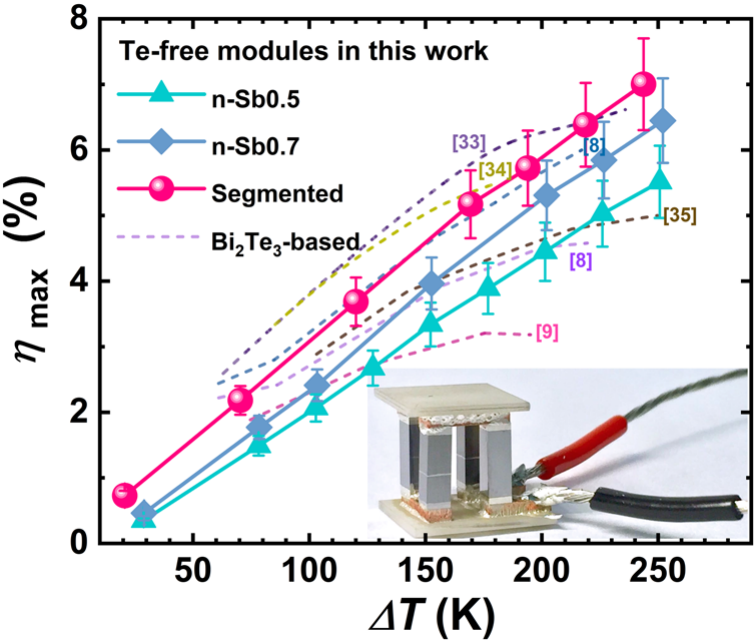
Performance of the Te-free segmented modules. Comparison of the measured conversion efficiency (*η*_max_, lines with symbols) among the Te-free modules in this work under a series of temperature difference (Δ*T*). The *η*_max_ of the Bi_2_Te_3_-based modules from the literature (dashed lines) were also plotted for comparison^8,9,33–35^. The inset shows the photograph of a segmented Te-free module. The error bars represent the measurement uncertainties from the commercial device.

Based on the simulation results, we assembled single-stage and segmented TE modules, each with 2 p-type and 2 n-type TE legs, with individual leg dimensions of 2 × 2 × 6.5 mm^3^. The p-type legs were MgAg_0.97_Sb_0.99_ for all modules, but the compositions of the n-type legs were altered in different modules, including single-stage n-Sb0.5 and n-Sb0.7, and segmented n-Sb0.5/Sb0.7. The output power (*P*), output heat flow (*Q*), and conversion efficiency of these modules were characterized as functions of the current (*I*) under a series of thermal loads (Δ*T*), in which the *T*_hot_ was varied from 323 K to 523 K and *T*_cold_ was maintained at ~293 K (Supplementary Fig. 5). As shown in Fig. 4, a high conversion efficiency (*η*_max_) of ~6.5% was realized in the single-stage modules for a temperature difference (Δ*T*) of 250 K. Moreover, the module with segmented legs boosted the *η*_max_ to ~7.0% for the same Δ*T*. The measured efficiencies were lower than the simulation results where the respective values are ~8.8% and ~9.3% for n-Sb0.7 single-stage and n-Sb0.5/Sb0.7 segmented module. As shown in the supporting information, the reduced measurement efficiency originates from an enlarged output heat flow, whereas the output power (Supplementary Figs. 3-4) were almost identical between simulations and measurements. The larger output heat flow in measurement suggests the potential existence of a thermal bypass, possibly due to the insufficient vacuum level or because of the direct thermal radiation from the hot side to the cold side in the Mini-PEM measurement setup since the small filling factor in our module (~16%). Our state-of-the-art modules are comparable to the Bi_2_Te_3_-based ones, and could potentially be improved by a better thermal management. In principle, our work demonstrated the feasibility of an Te-free TE modules for extended applications due to their remarkable sustainability.

## Discussion

Te-free TE modules could provide a clean and effective way of converting low-temperature waste heat to electricity. However, their practical applicability has been hindered by difficulties in synthesizing these Te-free materials on large scale, and high-performance TE modules based on such materials have not been successfully fabricated. We were able to synthesize high *zT* n-type Mg_3_(BiSb)_2_ and p-type MgAgSb by using a scalable routine that combined mechanical alloying and current-assisted sintering. By allowing a much simpler synthesis of such TE materials, our approach presents a substantial advance in shortening the synthesis period and reducing the elemental loss, which is especially essential for large-scale synthesis. The synthesized TE materials were subsequently translated into single-stage and segmented TE modules with conversion efficiency reaching ~6.5% and ~7.0%, respectively, for a temperature difference of ~250 K.

On the other hand, numerous challenges have to be overcome before realizing the ultimate substitution of Bi_2_Te_3_ module by a Te-free one, since the former has been investigated for more than half a century yet the latter is in its infancy. The required studies include but are not restricted to (1) upscale to the level of kilograms without degrading the TE properties, possibly by using planetary ball milling; (2) thermal cycle test to examine the device reliability at elevated temperatures; (3) long-term stability (in years) under actual operating conditions and different atmospheres such as current load and temperature gradient; (4) techniques for packing and sealing to overcome potential instabilities under atmosphere at elevated temperatures.

Despite the aforementioned challenges, this work thus realizes high-performance TE modules free from Bi_2_Te_3_ that are capable of harvesting low-grade (<550 K) waste heat. The efficiency demonstrated in this work exceeds that of the best Bi_2_Te_3_-based modules. Subsequent enhancements are possible upon further advance the material properties and optimize the filling factor of the modules. The use of abundantly available elements and the ease of fabrication render our modules a notable substitute for the Bi_2_Te_3_-based modules in low-grade-heat recovery. This will potentially spur the application of thermoelectric technology for power generation from low-grade heat.

## Methods

### Synthesis of n- and p-type materials

High-purity powder Bi (99.9%), Sb (99.99%), Te (99.99%), Mg (99.8%), and Ag (99.9%) were weighed out in the atomic ratios of Mg_3.3_Bi_1.498_Sb_0.5_Te_0.002_ (denoted as n-Sb0.5), Mg_3.3_Bi_1.298_Sb_0.7_Te_0.002_ (denoted as n-Sb0.7), and MgAg_0.97_Sb_0.99_ (denoted as p-MgAgSb). For each sample, the weighed elements were loaded into a hardened steel ball-milling jar in a glove box under an argon atmosphere with an oxygen and water level below 1.0 ppm and then ball-milled for 20 hours using a SPEX 8000D machine. The ball-milled powders were subject to field-assisted sintering (FAST, FCT System GmbH) together with the contact powders. Powders of iron (Fe, purity 99.8%) and silver (Ag, purity 99.9%) were selected as the contact layers for n-type and p-type materials, respectively. The n-type materials were sintered in a graphite die under a pressure of 50 MPa at 1023 K for 3 minutes, and the p-type materials were sintered in a tungsten carbide (WC) die under a pressure of 120 MPa at 553 K for 3 min.

The phase purity and crystal structure of the samples were examined by X-ray diffraction (XRD, Bruker D8, Co radiation) and their microstructures were analyzed by scanning electron microscopy (SEM). The sample homogeneity was characterized by energy-dispersive X-ray spectroscopy (EDX). The temperature-dependent Seebeck coefficient (*S*) and electrical conductivity (*σ*) were measured by the standard four-probe method (LSR-3, Linseis). The temperature-dependent thermal diffusivity (*λ*) was measured by a laser flash method under a helium atmosphere (LFA 1000, Linseis). The density (*ρ*) of the samples was measured by the Archimedes method, and the heat capacity (*C*_p_) was obtained from previous reports^17,20^. The thermal conductivity (*κ*_tot_) was calculated according to the relation *κ*_tot_ = *λ* ∙ *ρ* ∙ *C*_p_. The measurement uncertainties are 2%, 5%, and 7% for *σ*, *S*, and *κ*_tot_, respectively, which yield an error in *zT* of ~18%.

### Thermoelectric module fabrication and characterization

The sintered n- and p-type bulk samples with contact layers were cut into legs using a diamond wire saw in the dimension of 2.0 × 2.0 × 6.5 mm^3^. The TE legs (with the contacts), electrodes, and the ceramic substrates were bonded in a single step. The bonding was enabled by curing the silver paste at 548 K for 30 minutes in a high-vacuum tube furnace. The dimension of the module is 10 × 10 mm^2^ in cross-section and 9.3 mm in height as a combination of legs (6.5 mm), electrodes (0.8 mm × 2), and ceramic plates (0.6 mm × 2). Copper wires were soldered onto the cold-side electrodes for current and voltage measurements. The electrical output power (*P*) and the conversion efficiency (*η*) were measured under vacuum (< 5 × 10^−2^ Pa) by a Mini PEM (Advance Riko). To reduce the thermal contact loss, a graphite sheet (0.1 mm thickness) and thermal silicone grease (ST1002, Slont) were sandwiched between the module and the heater by a 60 N compression force. The hot-side temperature (*T*_hot_) of the thermoelectric element varied from 323 K to 548 K, whereas the cold-side temperature (*T*_cold_) was maintained at ~293 K. The radiative heat loss was compensated by a built-in program. An efficiency uncertainty of ~10% is applied based on the calibration results of a standard module that was provided by the company.

### Finite element simulation

COMSOL Multiphysics with Heat Transfer Module was used to perform the three-dimensional finite-element simulations of the power-generation characteristics for the thermoelectric module. A geometrical model with the same dimensions as the experimental thermoelectric element was used to calculate the electric power and heat flow outputs. Fourth-order polynomial fittings of the temperature-dependent Seebeck coefficient, electrical conductivity, and thermal conductivity for both n- and p-type materials were used as material properties in the simulations.

## Supplementary information

Supporting Information

## Data Availability

All data generated or analyzed during this study are included in the published article and its Supplementary Information. The data that support the findings of this study are available from the corresponding author upon reasonable request.
