# Supplementary material for: Towards tellurium-free thermoelectric modules for power generation from low-grade heat
Source: Nat Commun. 2021 Feb 18;12:1121. doi: 10.1038/s41467-021-21391-1 (PMC7892859; doi:10.1038/s41467-021-21391-1)
Supplement: Supplementary file 1 — Supporting Information [file 41467_2021_21391_MOESM1_ESM.pdf]

Towards tellurium-free thermoelectric modules for power generation from low-grade heat

Pingjun Ying[1#], Ran He[1#], Jun Mao[2], Qihao Zhang[1], Heiko Reith[1], Jiehe Sui[3], Zhifeng Ren[2*], Kornelius Nielsch[1,4,5*], Gabi Schierning[1*]

[1] *Leibniz Institute for Solid State and Materials Research, 01069 Dresden, Germany*

[2] *Department of Physics and Texas Center for Superconductivity at the University of Houston (TcSUH), University of Houston, Houston, TX 77204, USA*

[3] *National Key Laboratory for Precision Hot Processing of Metals, School of Materials Science and Engineering, Harbin Institute of Technology, 150001 Harbin, China*

[4] *Institute of Applied Physics, Technical University of Dresden, 01062 Dresden, Germany*

[5] *Institute of Materials Science, Technical University of Dresden, 01062 Dresden, Germany*

Supplementary Figures

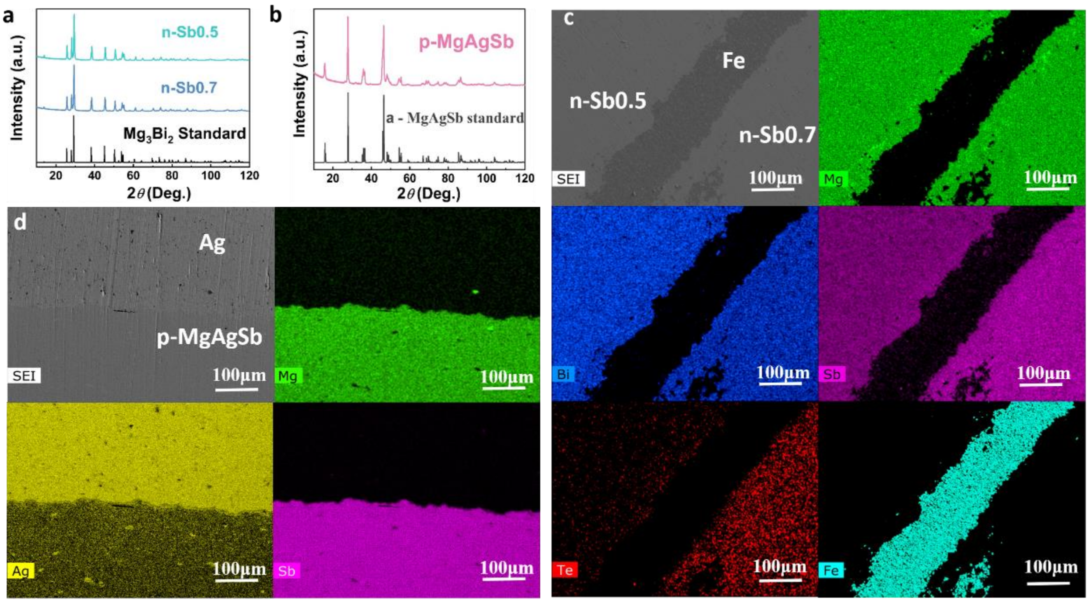

Supplementary Fig. 1. Sample characteristics. (**a, b**) XRD patterns of n-type and p-type samples. (**c, d**) SEM images of polished surfaces and corresponding elemental mapping for n-type and p-type samples with contact layers.

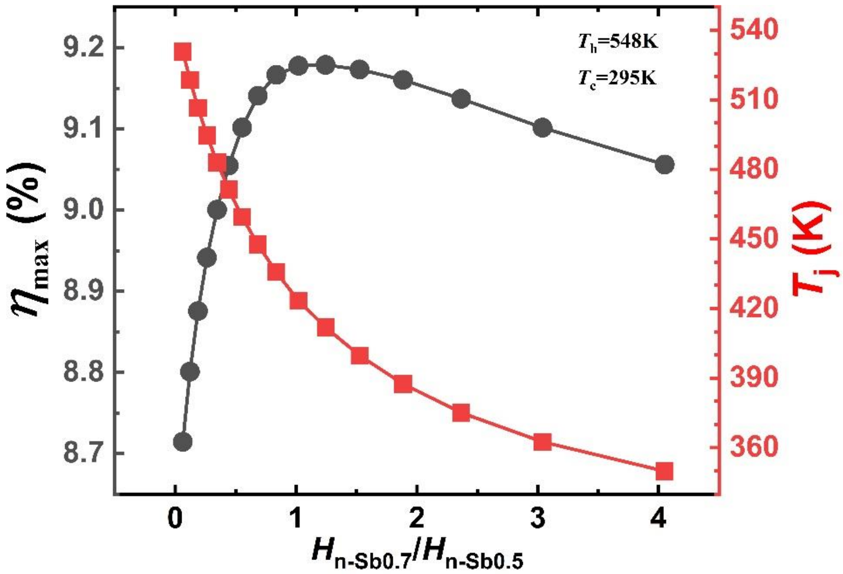

Supplementary Fig. 2. Simulation of the segmented module. Maximum conversion efficiency (η_{max}) and the junction temperature (T_j) between n-Sb0.5 and n-Sb0.7 as a function of the height ratio of $H_{n\text{-}Sb0.7}/H_{n\text{-}Sb0.5}$.

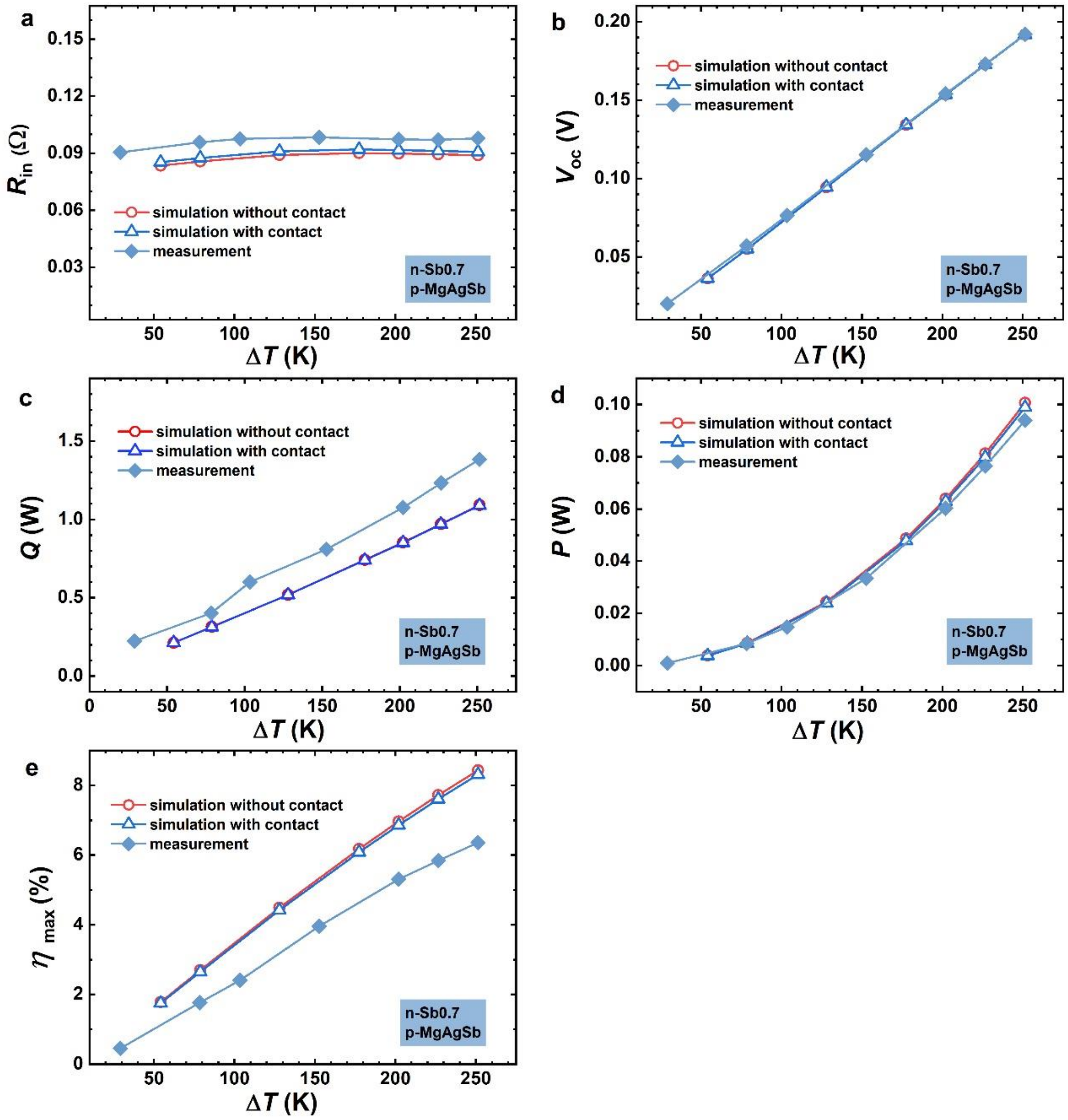

Supplementary Fig. 3. Comparison between simulated and measured data of module Sb0.7. Simulated and measured (**a**) internal resistance (R_{in}), (**b**) open-circuit voltage (V_{oc}), (**c**) output heat flow (Q), (**d**) output power (P) and (**e**) maximum conversion efficiency (η_{max}) with respect to a series of temperature difference (ΔT) of the single-stage module (n-Sb0.7) with and without contact resitance.

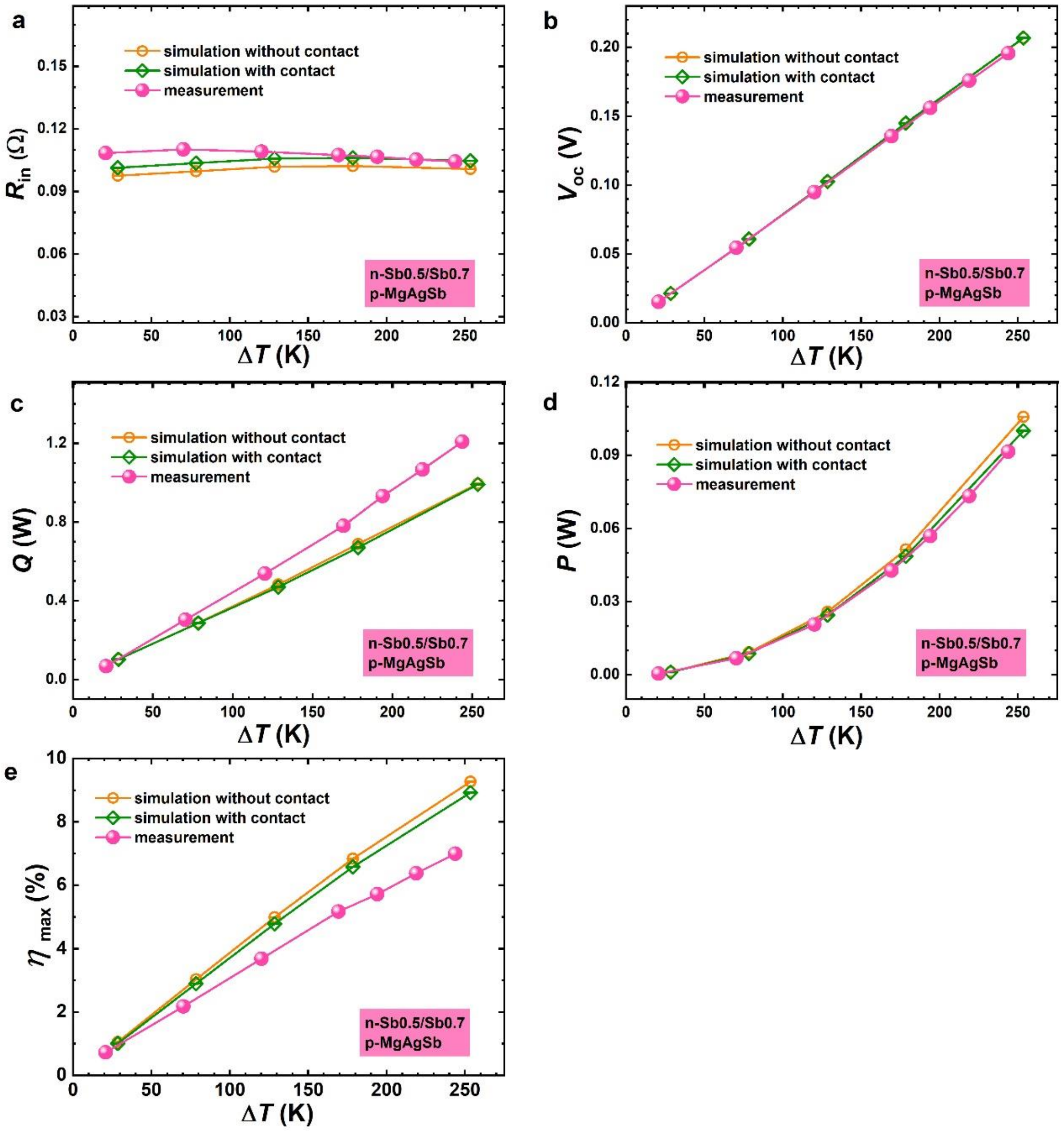

Supplementary Fig. 4. Comparison between simulated and measured data of segmented module. Simulated and measured (**a**) internal resistance (R_{in}), (**b**) open-circuit voltage (V_{oc}), (**c**) output heat flow (Q), (**d**) output power (P) and (**e**) maximum conversion efficiency (η_{max}) with respect to a series of temperature difference (ΔT) of the segmented module (n-Sb0.5/Sb0.7) with and without contact resitance.

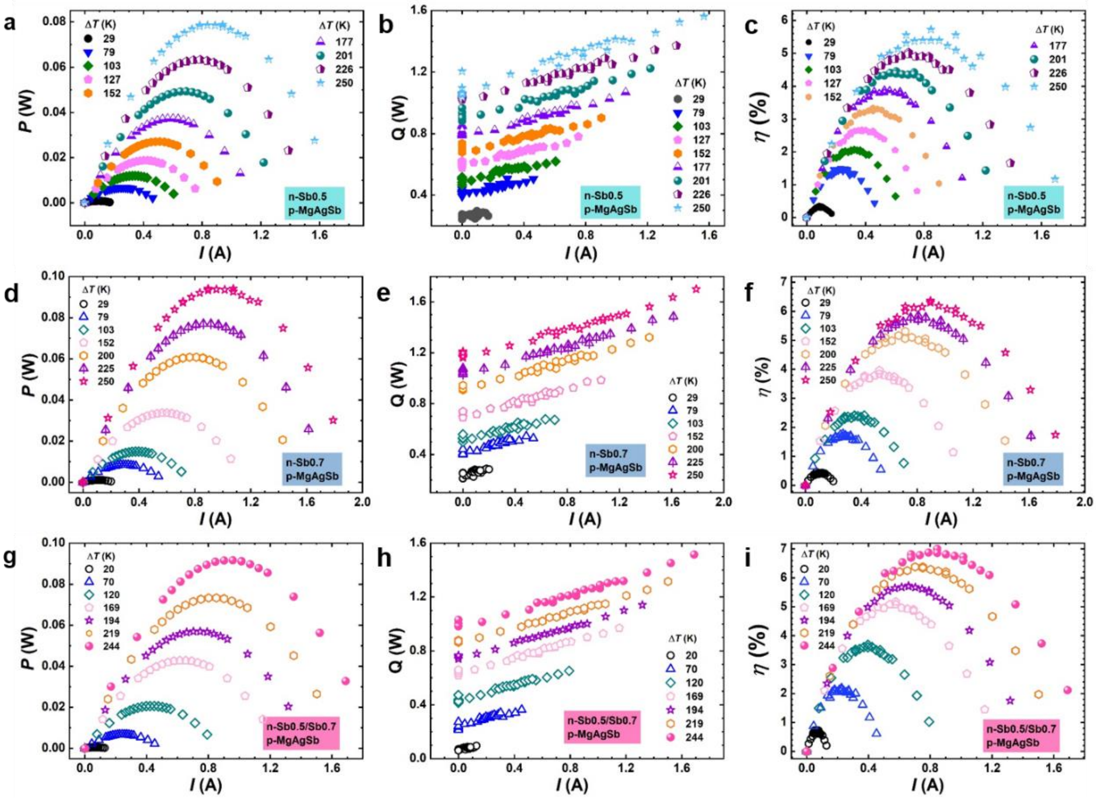

Supplementary Fig. 5. Performance of the Te-free TE modules. Measured current-dependent (**a, d, g**) Output power (*P*); (**b, e, h**) output heat flow (*Q*); and (**c, f, i**) conversion efficiency (η) of the Te-free modules with n-Sb0.5 (**a, b, c**), n-Sb0.7 (**d, e, f**), and segmented n-Sb0.5/Sb0.7 (**g, h, i**), respectively, under varied temperature differences.
